# Green Citrus Detection and Counting in Orchards Based on YOLOv5-CS and AI Edge System

**DOI:** 10.3390/s22020576

**Published:** 2022-01-12

**Authors:** Shilei Lyu, Ruiyao Li, Yawen Zhao, Zhen Li, Renjie Fan, Siying Liu

**Affiliations:** 1 College of Electronic Engineering (College of Artificial Intelligence), South China Agricultural University, Guangzhou 510642, China; lvshilei@scau.edu.cn (S.L.); 20203162097@stu.scau.edu.cn (R.L.); w844718456@163.com (Y.Z.); richard.fan@stu.scau.edu.cn (R.F.); 201934910820@stu.scau.edu.cn (S.L.); 2 Guangdong Laboratory for Lingnan Modern Agriculture, Guangzhou 510642, China; 3 Pazhou Lab, Guangzhou 510330, China; 4 Division of Citrus Machinery, China Agriculture Research System of MOF and MARA, Guangzhou 510642, China

**Keywords:** green citrus, object detection, virtual region, YOLOv5-CS, AI edge system

## Abstract

Green citrus detection in citrus orchards provides reliable support for production management chains, such as fruit thinning, sunburn prevention and yield estimation. In this paper, we proposed a lightweight object detection YOLOv5-CS (Citrus Sort) model to realize object detection and the accurate counting of green citrus in the natural environment. First, we employ image rotation codes to improve the generalization ability of the model. Second, in the backbone, a convolutional layer is replaced by a convolutional block attention module, and a detection layer is embedded to improve the detection accuracy of the little citrus. Third, both the loss function CIoU (Complete Intersection over Union) and cosine annealing algorithm are used to get the better training effect of the model. Finally, our model is migrated and deployed to the AI (Artificial Intelligence) edge system. Furthermore, we apply the scene segmentation method using the “virtual region” to achieve accurate counting of the green citrus, thereby forming an embedded system of green citrus counting by edge computing. The results show that the mAP@.5 of the YOLOv5-CS model for green citrus was 98.23%, and the recall is 97.66%. The inference speed of YOLOv5-CS detecting a picture on the server is 0.017 s, and the inference speed on Nvidia Jetson Xavier NX is 0.037 s. The detection and counting frame rate of the AI edge system-side counting system is 28 FPS, which meets the counting requirements of green citrus.

## 1. Introduction

The collection and monitoring of information throughout the fruit growth cycle essentially play a guiding role in the delicacy management of the fruit industry and has become the critical technical basis for constructing an intelligent orchard [1,2,3]. Recently, the application of computer vision technology for fruit detection has become a research hotspot. Especially, the detecting, tracking, and counting of green citrus are essential for predicting the yield of orchards during harvest. Research could provide reliable data support for orchard production management such as fruit thinning, sunburn prevention, and unmanned picking [4,5]. To realize the intelligent detection of fruits in the natural environment, researchers worldwide have successively explored and studied several solutions. Li et al. proposed an improved YOLOv3 lightweight model combined with the Mobile Net method for ripe fruit detection and applied it to dragon fruit detection in the actual environment [6]. Bi et al. used multiple segmentation methods to recognize citrus targets in the natural environment and improved multi-scale image detection and real-time performance of the citrus object detection model [7]. Xiong et al. applied the multi-scale convolutional neural network, the Des-YOLOv3 model, to realize the detection of citrus in the night environment [8]. Li et al. proposed the SSD-ResNet18 improved model to realize the real-time detection and classification of normal citrus, epidermal lesions, and mechanically damaged citrus [9]. Lyu et al. proposed the YOLOv3-LITE lightweight network model to realize real-time detection of mature citrus [10]. The colors of green fruits are similar to those of fruit tree leaves in tropical and subtropical evergreen orchards. Thus, Zheng et al. proposed the YOLO BP network to detect green citrus in the natural environment, and the results showed that the accuracy, recall, mean average precision (mAP), and detection speed of YOLO BP were 86%, 91% and 91.55% and 18 frames per second (FPS), respectively [11]. Kuznetsova et al. used YOLOv3 and YOLOv5 in general and close-up images. The average apple detection time was 19ms with FPR at 7.8% and FNR at 9.2% using YOLOv3 and with FPR at 3.5% and FNR at 2.8% using YOLOv5 [12]. Parico and Ahamed proposed a real-time pear fruit counter using YOLOv4 and DeepSORT, with an AP@0.50 of 98% [13]. Yan et al. proposed a light-weight fruit target real-time detection method for the apple picking robot based on improved YOLOv5, with an mAP of 86.75% [14]. Chen et al. proposed an improved YOLOv4 network structure to detect small citrus against a complex background, with an average increase in accuracy of 3.15% (from 92.89% to 96.04%) [15]. Xue et al. applied an improved YOLOv2 object detection model to accurately identify immature mangoes in an orchard environment [16]. Shi et al. also proposed a generalized attribution pruning detection method, which strips the sub-networks from large-scale networks for the real-time detection of mangoes [17]. Mu et al. performed transfer learning using a convolutional neural network, the R-CNN network, and Resnet-101 to detect immature tomatoes [18]. Wang et al. proposed an improved YOLOv5 model to detect small apples using the channel pruning method, with an average detection time of 8 ms per image. However, it was not applied to the mobile terminal for identification [19].

Presently, the majority of researchers focus on improving the static detection effect of different fruit targets [20,21,22]. However, related studies on the dynamic tracking and accurate counting of green citrus have seen less attention. Most citrus orchards in China are situated in hills and mountains, and the working environment of the orchards is complicated and changeable. Green citrus detection in orchards in the natural environment, tracking counting, and yield prediction of green citrus targets are more in line with the production needs of citrus orchards in China. In this paper, we adopted software and hardware co-design. We proposed an improved YOLOv5-CS (Citrus Sort) lightweight object detection model and migrated to the AI edge system platform to realize the intelligent detection of green citrus. Besides, using “virtual region”, scene segmentation was proposed to count the green citrus accurately. Thus, an embedded system for the intelligent detection of citrus orchards using edge computing was designed and realized.

## 2. Related Works

### 2.1. YOLO Models

**Figure 1:**
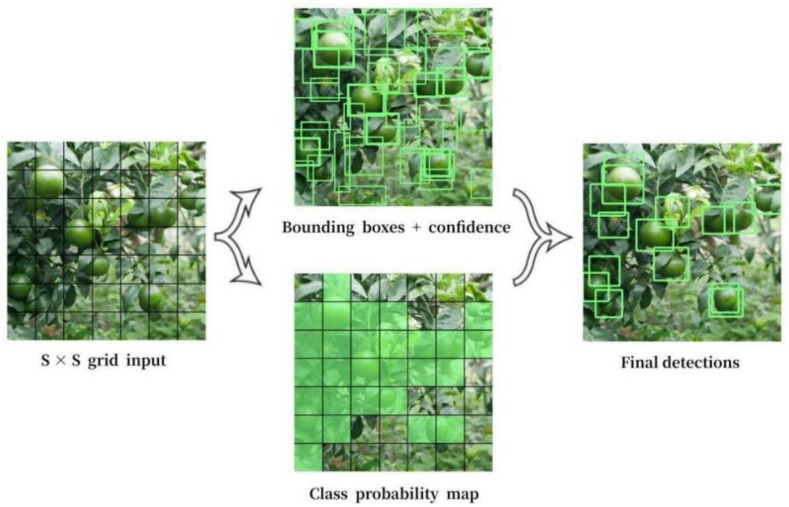
YOLO object detection.

Object detection, tracking and counting are vital techniques for realizing citrus fruit quantity statistics. According to the candidate region, object detection can be divided into two: single-stage object detector and two-stage object detector [23]. The single-stage object detector is an object detection model based on regression analysis, which omits the candidate region generation stage and directly obtains object classification and location information. The classic networks include YOLO, SSD, SqueezeDet and DetectNet. The first-level network of the two-stage object detector is used for the candidate region extraction. Candidate regions containing detection targets are selected from the input image, mainly through selective search or edge box model. The second-level network classifies the extracted candidate region and performs precise coordinate regression. The typical networks include a series of R-CNN models. Since the efficiency of the two-stage detection method using the candidate region does not meet real-time requirements and its computational cost is high, we proposed a YOLOv5-based single-stage detection model, theYOLOv5-CS model. In 2015, Redmon et al. proposed the YOLO model [24], which divided an image into S*S grids and by the center of the object in the grid on the prediction. However, its recall rate and detection accuracy are relatively low. Figure 1 shows a diagram of the YOLO object detection.

**Figure 2:**
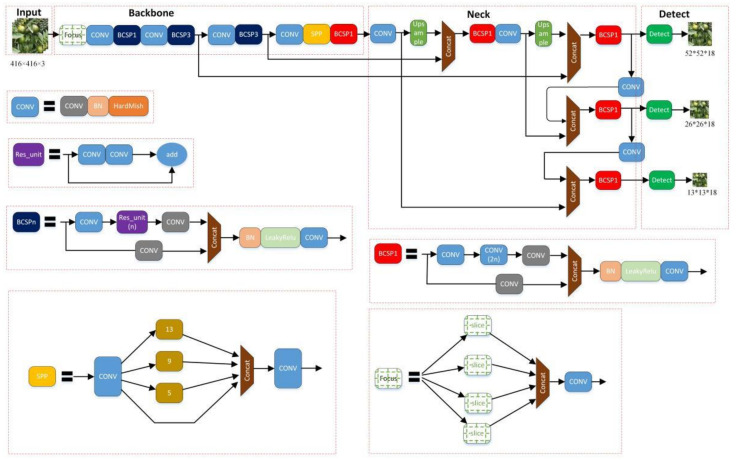
YOLOv5 Architecture.

In 2017, Redmon et al. proposed the YOLOv2 model [25], which uses the K-means clustering method to automatically select the best initial box, thereby improving the detection effect and speed compared to the previous version. Furthermore, in 2018, they proposed YOLOv3 and used the new Darknet-53 residual network for feature extraction, and three feature maps of different scales to predict [26]. In 2020, Bochkovskiy et al. proposed YOLOv4, which greatly improves the detection accuracy of the model [27]. Jocher proposed the YOLOv5 model in the same year, which is a lightweight network with a size of 27MB [28]. Here, we focus on the YOLOv5 model for detecting green citrus and other related research on quantitative statistics. The YOLOv5 model consists of four parts: the input layer, backbone network, neck network, and output detection layer. Figure 2 shows its network structure diagram.

#### 2.1.1. Input

YOLOv5 applied the Mosaic data augmentation method in the input layer. Then, it added a function to adjust the anchor box and the picture adaptively. In each training process, whether to adopt the adaptive adjustment of the anchor box and the image or not can be set for actual purposes. In this paper, we used different lengths and widths of the dataset pictures, so the size of the input pictures in the YOLOv5 network was uniformly modified to 416*416, thereby improving the inference and detection speeds. 

#### 2.1.2. Backbone

**Figure 3:**
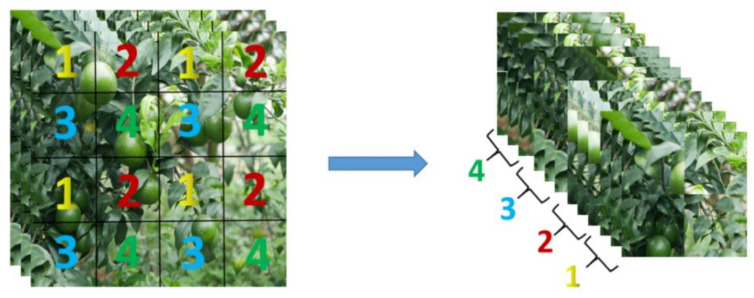
Slicing operation for subimage 1–4.

The focus module was applied to the backbone network of YOLOv5 to slice the input image. Take the unified image as an example, the image of 416*416*3 was put into the focus module to produce a picture of 208*208*12 after the slicing operation. After that, the image undergoes the convolution operation of 32 convolution kernels, and finally becomes a feature map of 208*208*32. Figure 3 shows the slicing operator of the focus module.

Additionally, the backbone network also includes the BCSP and SPP modules [29,30]. From Figure 2, blue BCSPn represents a module with a residual structure, and red BCSP1 represents a module without a residual structure. The BCSP module is used for improving the learning ability of the convolutional neural network, by making the model smaller while ensuring accuracy. This is conducive for the subsequent migration and deployment of the model on the AI (Artificial Intelligence) edge system. The SPP module is the spatial pyramid pooling module. The three pooling cores are 13*13, 5*5, and 9*9, respectively. The last one has no pooling operation but directly joins the Concat module with the other three channels and, finally, passes through Conv layer output. This module is beneficial for increasing the receptive field and the calculation speed does not decrease.

#### 2.1.3. Neck

**Figure 4:**
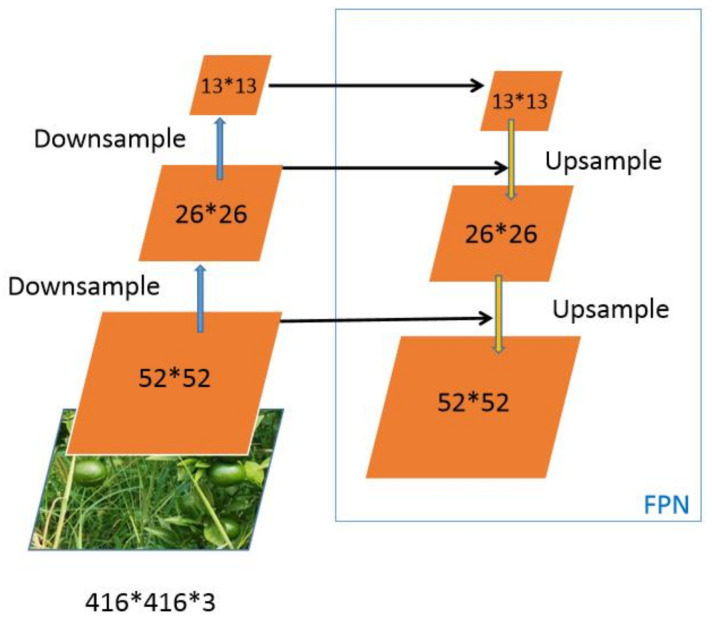
Neck module.

The Yolov5 neck network adopts an FPN structure [31], which is mainly used to generate feature pyramids and enhance the model’s ability to detect objects of different scales. Furthermore, it is used to recognize different sizes and standards of the same body. Figure 4 shows that after a couple of downsamples, the detected module outputs 52*52, 26*26, 13*13 respectively. 

#### 2.1.4. Output

Yolov5 used GIoU as the loss function of the bounding box. In the post-processing of target detection, Yolov5 used weighted NMS to filter target boxes.

### 2.2. Object Tracking and Counting

**Figure 5:**
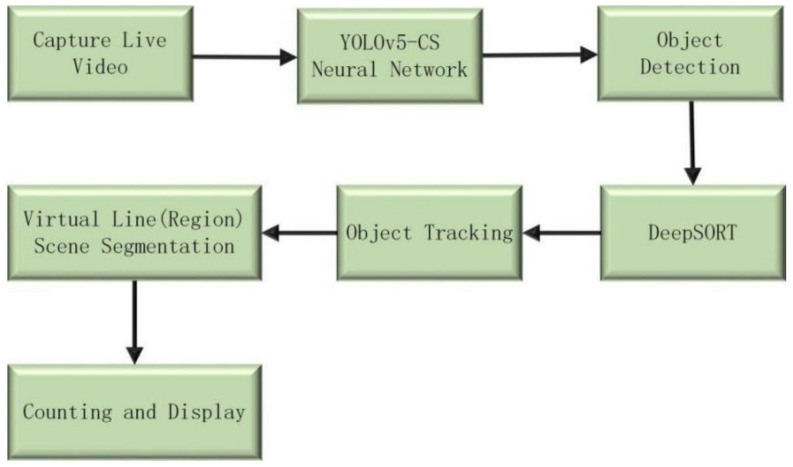
Green citrus counting.

DeepSORT had been proven to be one of the fastest and most robust methods for object tracking and counting [32]. It was originally developed by SORT and used object detection for tracking and effectively correlates the object detection of each frame [33]. When the position of an object changed in different frames, DeepSORT used the Hungarian algorithm to associate with the object in the previous frame [34], allowing the Kalman filter to predict the current position using the last location of the object [35]. Due to the similar size and color of citrus individuals, when the prediction box jumps, the ID of the prediction box represents citrus target changes, causing errors in quantitative statistics. Therefore, we introduced “virtual line” and “virtual region” to accurately predict the number of fruits of citrus fruit trees. Figure 5 shows the flowchart of green citrus counting.

**Figure 6:**
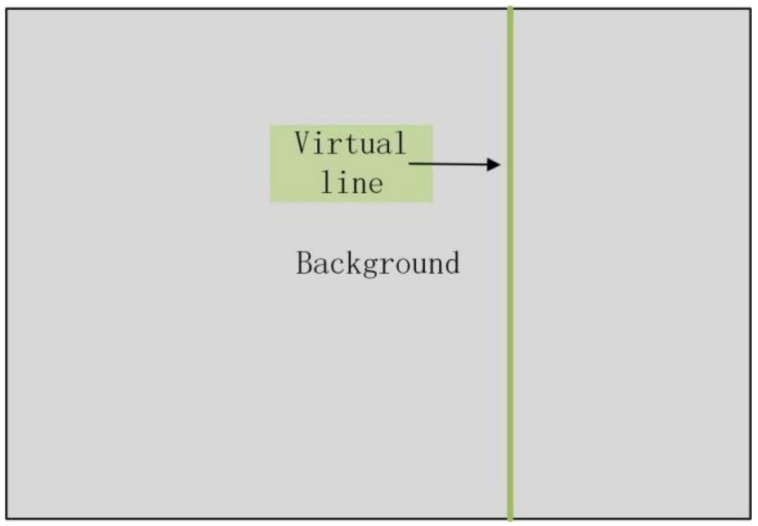
Virtual line for counting.

To count the number of green citrus, we set a virtual line in the video. This divides the scene and detects the center point of the citrus fruit prediction box. If the center point of the citrus prediction box crosses the virtual line, the number is increased by one. Therefore, we only need to walk around the tree so that the number of citrus can be counted. This method provides an efficient and accurate reference for citrus orchard yield estimation. Figure 6 shows the diagram of the virtual line for counting.

## 3. Materials and Methods

### 3.1. Dataset Preprocessing

#### 3.1.1. Data Acquisition

In this paper, we collected citrus images from citrus orchards in South China Agricultural University, Guangzhou Conghua and Hunan Yizhang, using DJI MAVIC Air2 drones, SLR cameras (Panasonic DMC-G7) and Honor 20 mobile phone for data collection. The shooting time was from 9:00 a.m. to 6:00 p.m. The shooting environment includes natural scenes, such as forward light, backlight, clear and blurred shots under sunny, cloudy, and rainy conditions. The shooting angles are forward, upward, overhead, and multiple angles. We collected more than 3000 original images.

#### 3.1.2. Data Augmentation and Labeling

**Figure 7:**
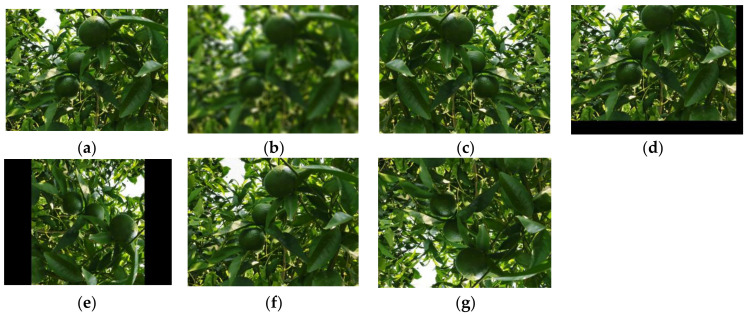
Data augmentation methods. (**a**) origin, (**b**) blur, (**c**) horizontal mirroring, (**d**) move, (**e**) rotate 270°, (**f**) noise, (**g**) vertical mirroring.

**Figure 8:**
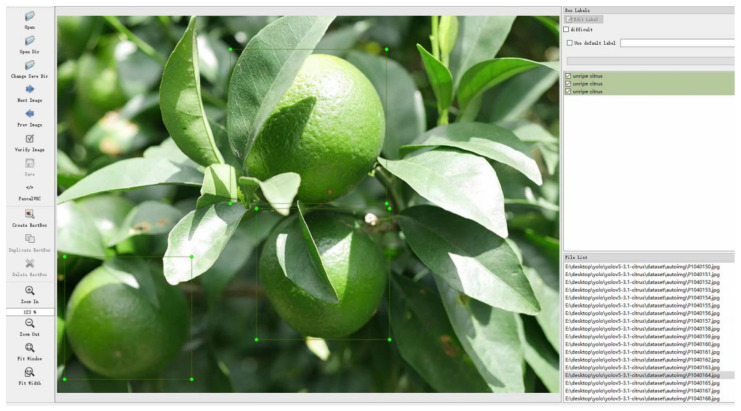
Data labeling.

The data were cleaned and filtered from more than 3000 original images and were augmented using data augmentation methods, such as vertical and horizontal mirroring, displacement, blur, rotation 270°, and salt and pepper noise, respectively. Figure 7 provides a view of these data enhancement methods. Finally, the original and augmented images are used as the dataset, containing 2831 images, such that the training and test sets are 2211 and 620, respectively, as shown in Table 1. Figure 8 shows the labeling process of the dataset using labeling software.

### 3.2. YOLOv5-CS Model Design and Edge-Computing System Migration & Deployment

#### 3.2.1. Model Design

**Figure 9:**
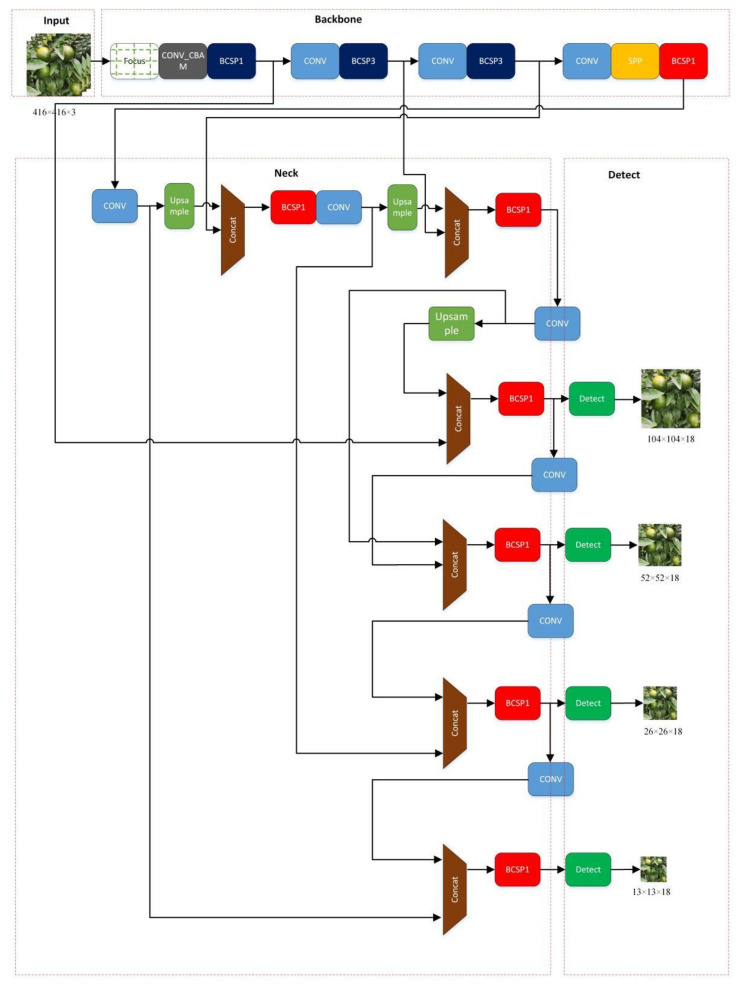
YOLOv5-CS Architecture.

**Figure 10:**
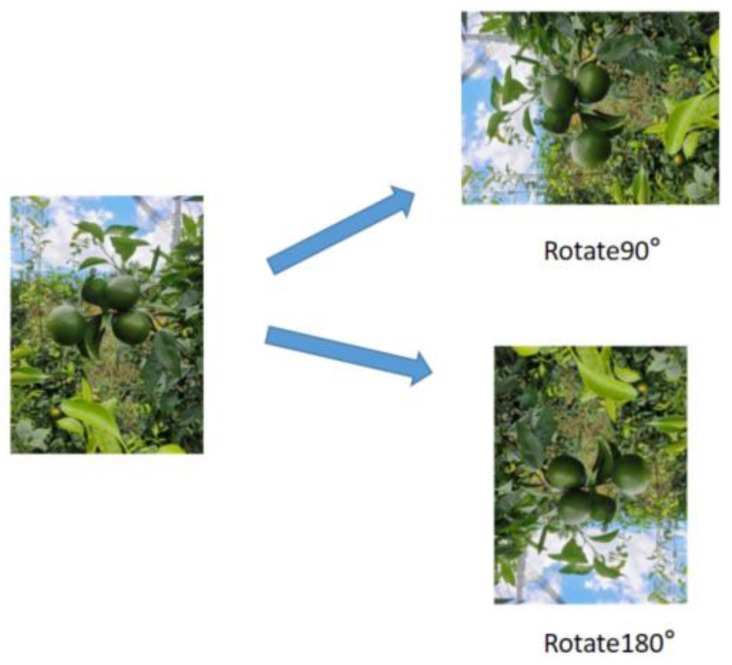
Image rotation.

Due to the difficulty of detecting green fruits in the natural environment and the time-consumption of manually taking statistics of citrus yields, this paper proposed a lightweight feasible detection and counting model using the improved YOLOv5-CS model and edge-computing platform. From Figure 9, the model used YOLOv5 as the main model, combined with the improved Conv_CBAM module to replace the original Conv module, which increased the model’s attention and feature extraction capabilities for different channels and spaces of the picture without increasing the calculation. Furthermore, it helped to improve the detection accuracy of green citrus. Additionally, Figure 10 shows a small target detection layer. It not only outputs the feature maps of 52*52, 26*26, and 13*13, but the output with a size of 104*104 strengthens the model to recognize small citrus fruits.

#### 3.2.2. Model Optimization

Because the green citrus is small with a color similar to the leaves, it is difficult for the YOLOv5 model to detect individual small citrus fruits. Thus, we improved the YOLOv5 by adding image rotation codes, a small object detection layer and an attention mechanism to improve the accuracy of the model for green citrus detection.

- Image Rotation

The YOLOv5 model provided many data augmentation methods. To improve the algorithm’s detection accuracy of green citrus from different angles, we added codes for vertical rotation at 90° and 180° to the model, effectively enhancing the model’s generalization ability. Figure 10 shows the image rotation.

- Small Object Detection Layer

Due to the size of the green citrus being relatively small, its pixel characteristics are similar to those of leaves under natural light. Therefore, adding modules to improve the detection accuracy and speed of the model is necessary. Thus, we added a small target detection layer and one more up-sampling and down-sampling process to the feature map of the YOLOv5 model. Simultaneously, the acquired feature map is Concat fused with that obtained in the second layer of the backbone network and so the obtained feature map with a larger size is used for small target detection. Table 2 shows the improved YOLOv5-CS network structure, where serial numbers 1 and 17–23 represent the replaced attention mechanism and additional small target detection module, respectively.

- CBAM

To detect the green citrus from the green leaves in Table 2, after the backbone network focus module, the attention mechanism CONV_CBAM module replaces the original CONV module to obtain more detailed information about the citrus and reduce interference from leaves and complex backgrounds. There are two attention mechanisms: squeeze-and-excitation (SE) and convolutional block attention module (CBAM). The SE module pays attention to the channel information, which mainly solves the loss problem caused by the different weights of different channels in the feature graph. The CBAM module includes both channel and spatial attention modules. The module takes the output of the channel attention module as input for the spatial attention module. After two pooling operations and a convolution operation with a convolution kernel of 7*7, the feature graph with the size of H*W*2 is obtained. Spatial attention features are also outputted through the Sigmoid function. The main innovation of the network is that the model can learn the spatial attention features of the output through the relationship between the channel and the space.

- Loss function

The loss function IoU is a commonly used evaluation indicator in target detection. It evaluates the distance between the predicted box B of the model and the ground truth of the model. The characteristic of IoU is that it is insensitive to scale. However, when both boxes B and $B^{\mathrm{gt}}$ do not overlap, the IoU value is zero. Here, there is no gradient return, and no learning or training can be performed.

(1) $$\mathrm{IoU}\;=\frac{\left|B\cap B^{\mathrm{gt}}\right|}{\left|B\cup B^{\mathrm{gt}}\right|},$$

(2) $$L_{\mathrm{IoU}}=1-\frac{\left|B\cap B^{\mathrm{gt}}\right|}{\left|B\cup B^{\mathrm{gt}}\right|},$$

where B denotes the predicted box and $B^{\mathrm{gt}}$ represents ground truth.

To solve this problem, Rezatofighi et al. proposed GIoU (generalized IoU) in 2019 [36]. GIoU focuses on areas where the predicted box overlaps the real box and other non-overlapping areas. When the prediction and real box do not overlap, the prediction box can be prompted to move toward the real box.

(3) $$\mathrm{GIoU}\;=\;\mathrm{IoU}\;-\frac{\left|\frac{C}{B\cup B^{\mathrm{gt}}}\right|}{\left|C\right|},$$

(4) $$L_{\mathrm{GIoU}}=1-\;\mathrm{IoU}\;+\frac{\left|C-B\cup B^{\mathrm{gt}}\right|}{\left|C\right|},$$

where C represents the smallest rectangular box in which B and $B^{\mathrm{gt}}$ can be contained. However, the method still has problems, such as an unstable prediction box and divergence of the training process. To directly minimize the distance between the two boxes for a better convergence, Zheng et al. proposed DIoU (Distance-IoU) and CIoU (Complete IoU) [37]. In contrast, DIoU introduces the distancing mechanism between the center points of the real and predicted boxes. For the horizontal and vertical directions, the DIoU loss converges quickly, and the GIoU loss remains almost the same as the IoU loss. However, the DIoU calculation does not consider the loss of width and height but only finds the overlapping area of the two boxes and the distance between both center points of the predicted and real boxes. However, if the center points of both boxes overlap but the width and height are different, the loss value is unchanged.

(5) $$\mathrm{DIoU}\;=\;\mathrm{IoU}\;-\frac{\rho^{2}\left(B,{\;B}^{\mathrm{gt}}\right)}{c^{2}},$$

(6) $$L_{\mathrm{DIoU}}=1-\;\mathrm{IoU}\;+\frac{\rho^{2}\left(B,{\;B}^{\mathrm{gt}}\right)}{c^{2}},$$

where represents the Euclidean distance between B and $B^{\mathrm{gt}}$, and c represents the length of the diagonal line containing the smallest box. On this basis, they proposed CloU. CIoU adds an impact factor, which considers the aspect ratio of the predicted box to fit the real box.

(7) $$L_{\mathrm{CIoU}}=1-\;\mathrm{IoU}\;+\frac{\rho^{2}\left(B,{\;B}^{\mathrm{gt}}\right)}{c^{2}}+\;\alpha \nu ,$$

(8) $$\nu \;=\frac{4}{\pi^{2}}{\left(\tan^{-1}\frac{w^{\mathrm{gt}}}{h^{\mathrm{gt}}}-\tan^{-1}\frac{w}{h}\right)}^{2},$$

(9) $$\alpha \;=\frac{\nu }{\left(1-\mathrm{IoU}\right)+\nu },$$

where α is a positive tradeoff parameter, and ν measures the consistency of the aspect ratio.

#### 3.2.3. Edge-Computing System Migration & Deployment

**Figure 11:**
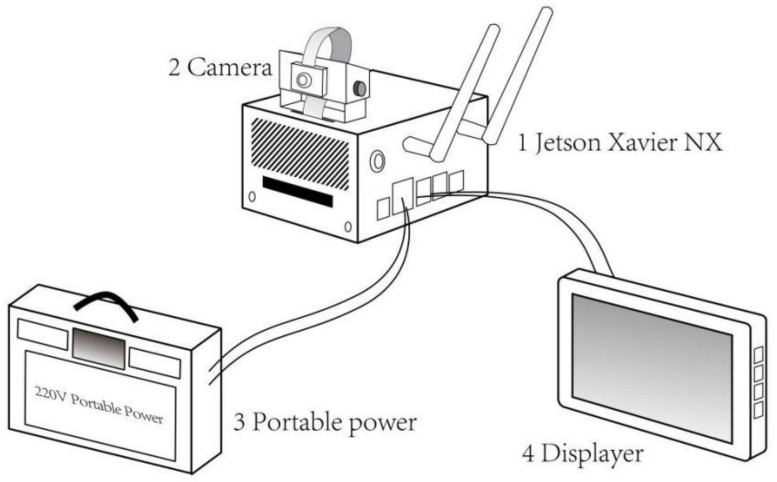
Edge-computing system platform.

The Jetson Xavier NX edge-computing platform based on ARM architecture and produced by NVIDIA was applied in this paper. The platform is small, approximately 70 × 45 mm in size, and brings the performance of supercomputers to the edge through the system of modules. The platform includes a 6-core Carmel ARM CPU, 384 NVIDIA CUDA^®^ Cores, 48 Tensor Cores, and two NVIDIA deep learning accelerator (NVDLA) engines, which can provide up to 21 TOPS of computing power. Camera, displayer, and portable power devices were added to form an edge-computing system platform based on the edge-computing platform. Figure 11 shows the edge-computing system platform diagram.

**Figure 12:**
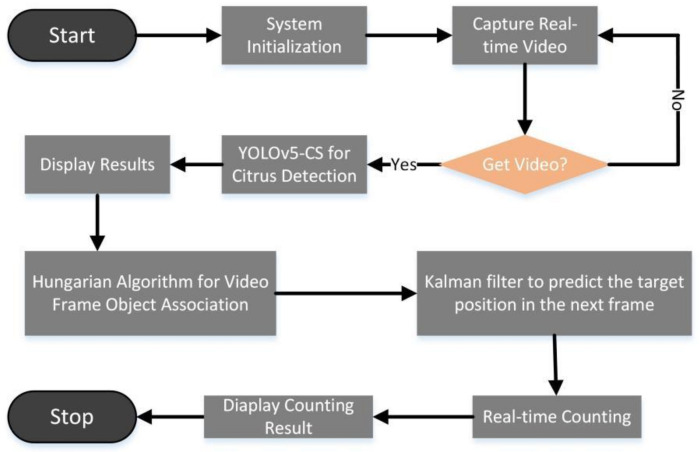
Green citrus counting.

To facilitate the deployment of the YOLOv5-CS model, it is necessary to deploy the operating environment on Jetson Xavier NX and call the hardware performance of the platform, especially the deep learning accelerator engine. To achieve production statistics, USB and HDMI interfaces and expansion pins of the platform were used. The flowchart of counting the number of green citrus is shown in Figure 12. The edge-computing platform obtained the video stream in a short time by calling the high-definition camera and transmitting the video stream information into the RAM. The CPU control modules, such as CUDA cores, Tensor cores, and NVDLA, use heterogeneous parallel computing to accelerate the model by hardware. The detection result, as well as the number of citrus, were displayed using the visualization module.

### 3.3. Learning Rate

The learning rate affects the convergence speed of the YOLOv5-CS model. Here we used the cosine annealing algorithm to dynamically change the learning rate. In the warm-up stage, one-dimensional linear interpolation was used to update the learning rate of each iteration, after which the cosine annealing algorithm was used to update the learning rate. The cosine annealing mechanism uses a cosine function to reduce the learning rate. This descent mode is matched with the learning rate. Increasing the learning rate can effectively prevent the model from falling into the local minimum and then train in the direction of the global minimum.

(10) $$\eta_{t}={\;\eta }_{\min}^{i}+\frac{1}{2}(\eta_{\max}^{i}-{\;\eta }_{\min}^{i})\left(1+\cos(\frac{T_{\mathrm{cur}}}{T_{i}}\pi \right)),$$

where $\eta_{\min\;}^{i}$ and $\eta_{\max}^{i}$, respectively, represent the minimum and maximum of the learning rate, and define the learning rate range. $T_{\mathrm{cur}}$ denotes the number of epochs executed since the last training restart, although it is updated after each batch is run. When an epoch has not been executed, the value of $T_{\mathrm{cur}}$ can be a decimal. $T_{i}$ denotes the total number of epochs in the i-th training session.

### 3.4. Experiment Platform

Server-side: Windows 10, Core i9-11900@5.20 GHz CPU, 32 GB RAM, NVIDIA GeForce RTX 3080(10 GB) GPU. AI edge system side: 6-core NVIDIA Carmel ARM^®^v8.2 64-bit CPU. The model framework is Pytorch, with related software CUDA 11.1, cudnn 11.1, and Python 3.8.10. The original YOLOv5 model and the improved YOLOv5-CS model use YOLOv5s.pt for pretraining and retraining based on the pretraining results. The parameters are set as follows: image input size: 416*416, epoch: 50, initial learning rate: 0.01, bbox_iou: CIoU.

### 3.5. Evaluation Index

Since the background environment can be identified as citrus or missed during the detection, the accuracy and recall ratios are used to describe the citrus detection. The accuracy and recall rates are given as follows:

(11) $$\mathrm{Precision}\;=\frac{\mathrm{TP}}{\mathrm{TP}+\mathrm{FP}},$$

(12) $$\mathrm{Recall}\;=\frac{\mathrm{TP}}{\mathrm{TP}+\mathrm{FN}},$$

where TP and FP denote positive and negative samples, respectively, predicted to be true, and FN represents a positive sample predicted to be false. By setting the accuracy and recall rate to the vertical and horizontal axes, respectively, gives the accuracy–recall rate curve, referred to as the P-R curve.

To better evaluate the effect of the model, the AP (average precision) of a single category was proposed as the sum of the AP values of each category, which is used to obtain mAP (mean average precision). The definitions of AP and mAP are as follows:

(13) $$\mathrm{AP}\;=\int_{0}^{1}P\left(R\right)\mathrm{dR},$$

(14) $$\mathrm{mAP}\;=\frac{\sum_{q=1}^{Q}\mathrm{AP}\left(q\right)}{Q},$$

where Q is the number of categories.

## 4. Experiment and Results

### 4.1. Ablation Experiment

To prove the effectiveness of our model in this paper, we conducted the ablation experiment on both data augmentation processing methods and improved architecture, as shown in Table 3. √ represents using the methods or module.

From the table above, it can be seen that the detection accuracy was improved from 96.66% to 97.51% by using data augmentation methods, and the recall was improved by 3.42%. After adding the small object detection layer, the detection accuracy reached 97.59%. The detection accuracy of the model finally achieved 98.05% after adding CBAM.

### 4.2. Training Result

**Figure 13:**
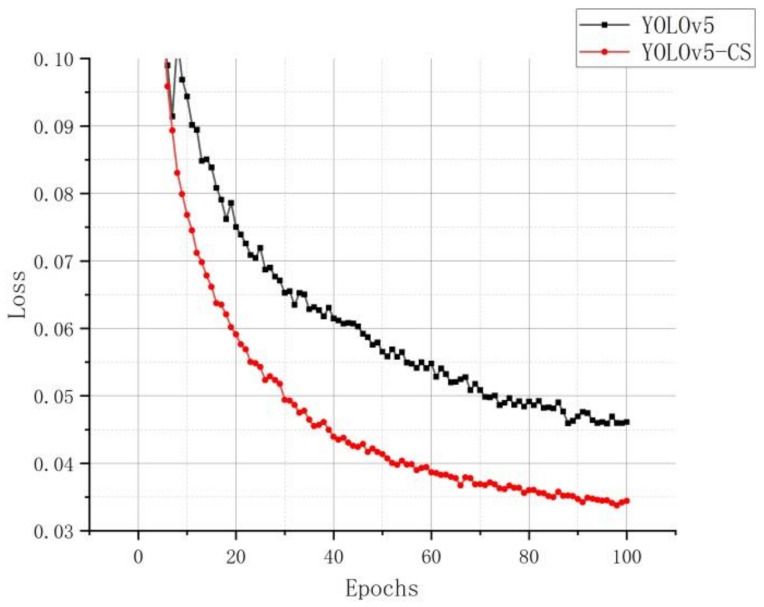
Loss curve.

The performance of the detection model is evaluated using the loss function curve and the mAP@.5. The loss function curve shows the network model’s convergence speed and degree of convergence during the training process. Figure 13 shows the loss function curve of the YOLOv5 and improved YOLOv5-CS models.

**Figure 14:**
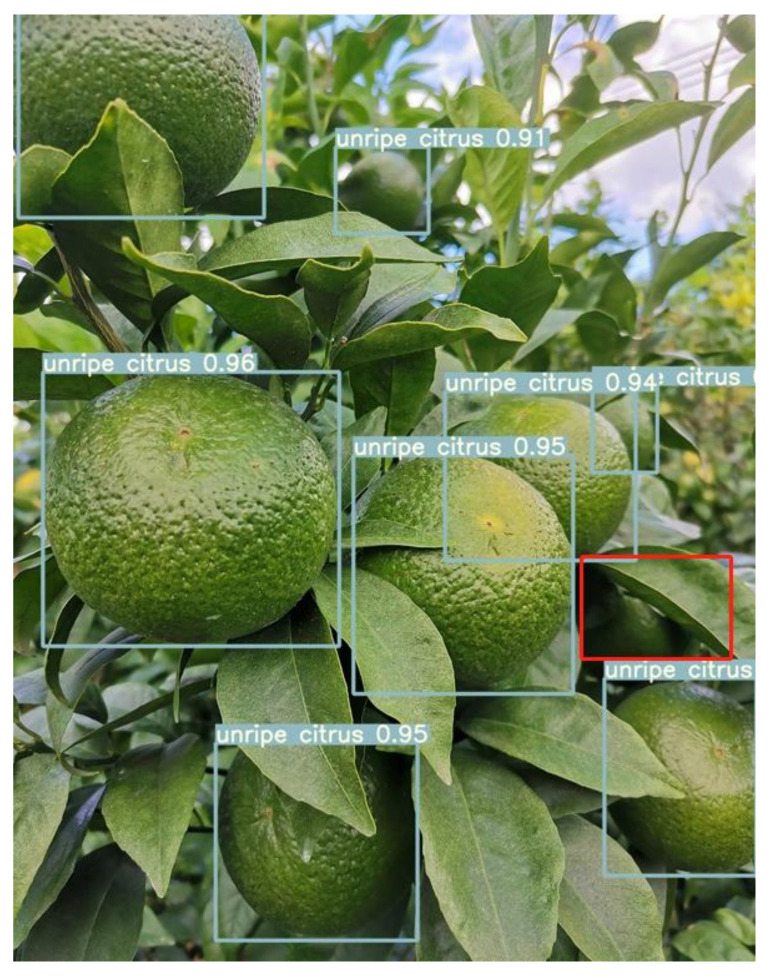
Leakage detection of citrus.

From the figure above, the improved YOLOv5-CS model curve converged with a faster degree of convergence and a smaller loss value in the training process of 100 epochs. When it finished, the loss value of the improved YOLOv5-CS model was 21.74% lower than that of the YOLOv5 model, which proves that the former has better convergence. Although the loss value has achieved good results, there are still some pictures in the test set that are missed or encountered errors due to excessive occlusion and light, as shown in Figure 14.

**Figure 15:**
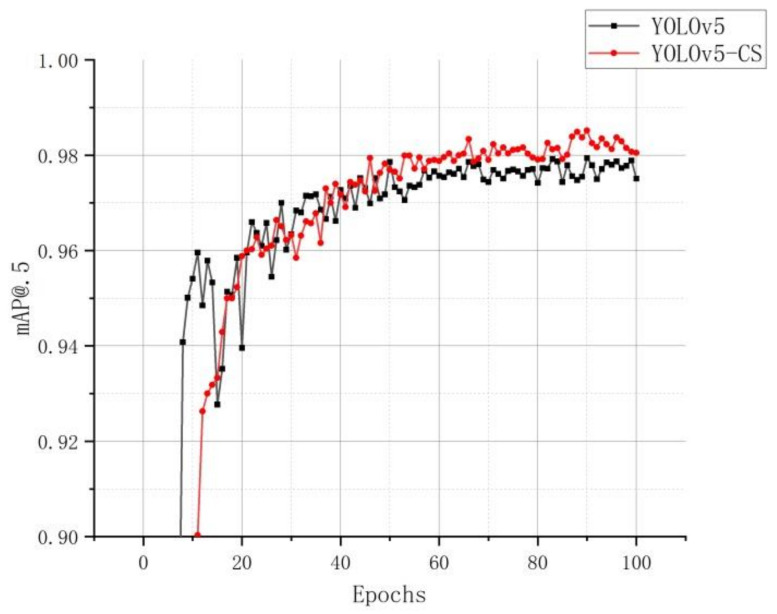
mAP@.5 curve.

mAP@.5 was used to measure the detection effect of the model. The higher its value, the better the detection effect of the model. Figure 15 shows that, after 100 epochs, the mAP@.5 value of the improved YOLOv5-CS model gradually stabilized, reaching 98.05%, and the highest during the period reached 98.5%. This shows that the improved YOLOv5-CS model has a better result for detecting green citrus and has reached the expectation of accurate recognition.

The improved YOLOv5-CS model and the YOLOv5 model were trained for the first time with 100 epochs, respectively, and the improved YOLOv5-CS model was then retraining with 50 epochs; the parameters obtained are shown in Table 4. Although the precision value of the improved YOLOv5-CS model decreased, the final values of mAP@.5 and recall were 98.23% and 97.66%, respectively, and the detection time for 620 images in the validation set was 1.61 s less than that of the YOLOv5 model.

### 4.3. Counting Result

**Figure 16:**
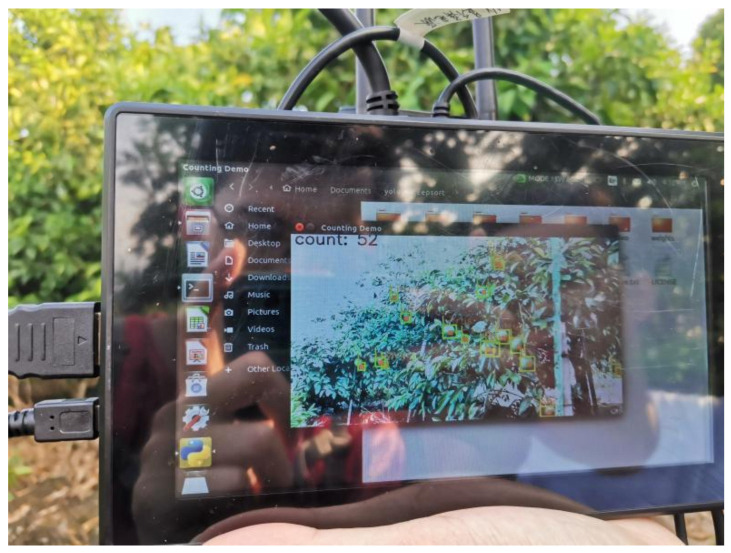
Counting the number of citrus in the orchard.

By setting a virtual line on the image screen, the citrus tracked and identified will be detected and counted when passing the line. However, the citrus prediction box may flicker when passing through the line; therefore, to accurately count the number of citrus, we expand the virtual line into a virtual region to ensure citrus detection through this area and improve the accuracy of citrus yield statistics. The diagram of the virtual region in the orchard environment is shown in Figure 16.

We conducted experiments on the simulated citrus trees and the real citrus trees. Standing at a distance of 1.5 m from the citrus trees, the camera was located at half the height of each citrus tree. The counting results of citrus are shown in Table 5 and Table 6. The average relative error of the count of the simulated citrus tree was 4.25%, and the average relative error of the count of the real citrus tree was 8.75%. The main reason of the different errors mainly came from the complexity of the orchard environment, which caused false detection.

## 5. Discussion

In this paper, we proposed the YOLOv5-CS model with improved robustness and stability in a complex natural orchard environment using the data augmentation method. Additionally, we applied the CBAM module to make the model focus on important feature information and ignore unimportant information. Furthermore, a detection layer was embedded to improve the model’s detection accuracy for little citruses. Additionally, using the CIoU as the loss function, it achieved the fastest convergence speed and best convergence effect function. The cosine annealing algorithm was applied to change the learning rate when the model fell into the local optimal solution, thereby training it towards the global optimal solution. Furthermore, the retraining method was used to improve the mAP@.5 and recall values of the model, thereby improving the convergence speed of the model. These improvement methods produced excellent detection results. The inference speeds of YOLOv5s and YOLOv5-CS detecting a picture on the server were 0.018 s and 0.017 s respectively, and the inference speeds of those on the Nvidia Jetson Xavier NX were 0.04 s and 0.037 s respectively. Next, we used DeepSORT for citrus object tracking and counting. This involved tracking the location of citrus targets by combining the Kalman filtering with the Hungarian algorithm for frame-by-frame analysis of the video. Additionally, we introduced “virtual line” and “virtual region” to improve the accuracy of the citrus target count of the model and avoid duplicate counts. We also migrated the model to the Jetson Xavier NX edge-computing platform to help farmers count the number of green citrus. High-definition cameras were used to capture videos and showed the counting result on a visualization module. Finally, we realized the feasible detection of green citrus and counting on the mobile platform.

If the green citrus detection and counting model could apply to the entire orchard, the rich dataset will help identify citrus at different growth stages and collect several fruits, which can construct various fruits detection and counting systems for detecting and counting different fruits simultaneously. Hence, our system will have more functional applications in the intelligent orchard.

## 6. Conclusions and Future Works

Recently, there has been rapid development in object detection and multi-object tracking with great interest in their applications in agriculture. Briefly, the contributions of this paper are as follows. Here, an improved YOLOv5-CS model was proposed as an extension of the YOLOv5 model. Our model adopted object detection and multi-object tracking technology, to achieve feasible detection and counting of green citrus in orchards. Codes for image rotation were added to data augmentation. The CBAM module and the small-object detection layer were embedded in the backbone network. The CIoU loss function and cosine annealing algorithm were introduced in the training. We migrated the improved YOLOv5-CS model to the edge-computing platform to help farmers use mobile devices to detect and count green citrus, which is of great reference significance for citrus yield estimation. Until now, this is the first time in the computer field that the object detection and counting of citrus were applied to the edge-computing platform and the “virtual region” was introduced to realize the counting of green citrus in the orchard. Compared with the original YOLOv5 model, the mAP@.5 and recall values of the improved model improved by 0.72% and 1.50%, respectively. The inference speeds of YOLOv5s and YOLOv5-CS detecting a picture on the server were 0.018 s and 0.017 s respectively, and the inference speeds of those on Nvidia Jetson Xavier NX were 0.04 s and 0.037 s respectively. The detection and counting frame rate of the video on the edge-computing platform was 28 FPS. It also had strong robustness in the complex orchard environment, providing farmers with portable and intelligent citrus counting equipment, thus reducing planting costs caused by manual counting. In the future, we will continue to improve the prediction accuracy and speed, especially that of citrus at different growth stages. Nowadays, yield estimation of fruits and automated picking have become important development directions for orchard management and production. We will also study the deployment of citrus counting equipment on drones, study autonomous navigation and synchronized positioning using SLAM and visual navigation technologies, and contribute to the development of intelligent orchards.

## Figures and Tables

**Table 1:** Green citrus dataset.

Dataset	Label	Training Set	Test Set	Total
green citrus	unripe citrus	2211	620	2831

**Table 2:** YOLOv5-CS network structure.

Serial Number	From	Params	Module	Arguments
0	−1	3520	Focus	[3, 32, 3]
**1**	**−1**	**19,170**	**Conv_CBAM**	**[32, 64, 3, 2]**
2	−1	19,904	BottleneckCSP	[64, 64, 1]
3	−1	73,984	Conv	[64, 128, 3, 2]
4	−1	161,152	BottleneckCSP	[128, 128, 3]
5	−1	295,424	Conv	[128, 256, 3, 2]
6	−1	641,792	BottleneckCSP	[256, 256, 3]
7	−1	1,180,672	Conv	[256, 512, 3, 2]
8	−1	656,896	SPP	[512, 512, [5, 9, 13]]
9	−1	1,248,768	BottleneckCSP	[512, 512, 1, False]
10	−1	131,584	Conv	[512, 256, 1, 1]
11	−1	0	Upsample	[None, 2, ‘nearest’]
12	[−1, 6]	0	Concat	[1]
13	−1	378,624	BottleneckCSP	[512,256, 1, False]
14	−1	66,048	Conv	[256, 128, 1, 1]
15	−1	0	Upsample	[None, 2, ‘nearest’]
16	[−1, 4]	0	Concat	[1]
**17**	**−1**	**345,856**	**BottleneckCSP**	**[384, 256, 1, False]**
**18**	**−1**	**33,024**	**Conv**	**[256, 128, 1, 1]**
**19**	**−1**	**0**	**Upsample**	**[None, 2, ‘nearest’]**
**20**	**−1**	**0**	**Concat**	**[1]**
**21**	**−1**	**86,912**	**BottleneckCSP**	**[192, 128, 1, False]**
**22**	**−1**	**147,712**	**Conv**	**[128, 128, 3, 2]**
**23**	**−1**	**0**	**Concat**	**[1]**
24	−1	95,104	BottleneckCSP	[256, 128, 1, False]
25	−1	147,712	Conv	[128, 128, 3, 2]
26	[−1, 14]	0	Concat	[1]
27	−1	313,088	BottleneckCSP	[256,256, 1, False]
28	−1	590,336	Conv	[256, 256, 3, 2]
29	[−1, 10]	0	Concat	[1]
30	−1	1,248,768	BottleneckCSP	[512,512, 1, False]

**Table 3:** Model ablation experiment.

Data Augmentation	Small Object Detection Layer	CBAM	mAP@.5	Recall	Epochs
			96.66%	92.74%	100
√			97.51%	96.16%	
√	√		97.59%	96.09%	
√	√	√	98.05%	97.38%	

**Table 4:** Training result.

Neural Network	Epochs	mAP@.5	Image-Size	Precision	Recall
First training					
YOLOv5	100	97.51%	416	89.81%	96.16%
YOLOv5-CS	100	98.05%	416	84.49%	97.38%
Retraining					
YOLOv5	50	97.79%	416	93.03%	95.27%
YOLOv5-CS	50	98.23%	416	86.97%	97.66%

**Table 5:** Counting results of simulated citrus tree.

Number of Experiments	Actual Number	Number of Tests	Relative Error	Average Relative Error of Ten Times	FPS
1	40	40	0%	4.25%	28
2		37	7.5%		
3		36	10%		
4		38	5%		
5		38	5%		
6		39	2.5%		
7		37	7.5%		
8		39	2.5%		
9		40	0%		
10		39	2.5%		

**Table 6:** Counting results of real citrus tree.

Number of Experiments	Actual Number	Number of Tests	Relative Error	Average Relative Error of Ten Times	FPS
1	24	22	8.33%	8.75%	28
2		21	12.5%		
3		23	4.17%		
4		25	4.17%		
5		21	12.5%		
6		20	16.67%		
7		26	8.33%		
8		22	8.33%		
9		23	4.17%		
10		22	8.33%		

## Data Availability

Not applicable.
